# Involvement of FGFR4 Gene Variants on the Clinicopathological Severity in Urothelial Cell Carcinoma

**DOI:** 10.3390/ijerph17010129

**Published:** 2019-12-23

**Authors:** Ming-Dow Tsay, Ming-Ju Hsieh, Chia-Yi Lee, Shian-Shiang Wang, Chuan-Shu Chen, Sheng-Chun Hung, Chia-Yen Lin, Shun-Fa Yang

**Affiliations:** 1 Institute of Medicine, Chung Shan Medical University, Taichung 402, Taiwan; tmd04834@gmail.com (M.-D.T.); 170780@cch.org.tw (M.-J.H.); sswdoc@vghtc.gov.tw (S.-S.W.); r2060d@yahoo.com.tw (C.-S.C.); weshong1118@gmail.com (S.-C.H.); lcyhank.tw@gmail.com (C.-Y.L.); 2 Department of Family medicine, Tungs’ Taichung MetroHarbor Hospital, Taichung 433, Taiwan; 3 Cancer Research Center, Changhua Christian Hospital, Changhua 500, Taiwan; 4 Graduate Institute of Biomedical Sciences, China Medical University, Taichung 404, Taiwan; 5 Department of Ophthalmology, Show Chwan Memorial Hospital, Changhua 500, Taiwan; ao6u.3msn@hotmail.com; 6 Department of Optometry, College of Medicine and Life Science, Chung Hwa University of Medical Technology, Tainan 717, Taiwan; 7 School of Medicine, Chung Shan Medical University, Taichung 402, Taiwan; 8 Division of Urology, Department of Surgery, Taichung Veterans General Hospital, Taichung 407, Taiwan; 9 Department of Applied Chemistry, National Chi Nan University, Nantou 545, Taiwan; 10 Department of Medical Research, Chung Shan Medical University Hospital, Taichung 402, Taiwan

**Keywords:** fibroblast growth factor receptor 4, single-nucleotide polymorphism, urothelial cell carcinoma, tumor stage

## Abstract

Fibroblast growth factor receptor 4 (FGFR4) plays a prominent role in cell proliferation and cancer progression. This study explored the effect of FGFR4 single-nucleotide polymorphisms (SNPs) on the clinicopathological characteristics of urothelial cell carcinoma (UCC). This study was conducted to survey the possible correlation of the polymorphism of FGFR4 to the risk and clinicopathologic characteristics of UCC. Four loci of FGFR4 (rs2011077 T > C, rs351855 G > A, rs7708357 G>A, and rs1966265 A > G) were genotyped via the TaqMan allelic discrimination approach in 428 UCC cases and 856 controls. The results indicated that UCC subjects who carried the SNP rs2011077 TC+CC genotypes were significantly related to a higher tumor stage (odds ratio (OR): 1.751, 95% confidence interval (CI): 1.078–2.846), primary tumor size (OR: 1.637, 95% CI: 1.006–2.662), and histopathologic grading (OR: 1.919, 95% CI: 1.049–3.511). Moreover, the SNP rs1966265 AG+GG genotypes were prominently related to a higher tumor stage (OR: 1.769, 95% CI: 1.082–2.891), primary tumor size (OR: 1.654, 95% CI: 1.011–2.706), and histopathologic grading (OR: 2.006, 95% CI: 1.096–3.674) compared to individuals with AA homozygotes. In conclusion, our data reveal association of FGFR4 polymorphisms with UCC clinicopathologic characteristics. FGFR4 polymorphisms may serve as a marker or therapeutic target in UCC development.

## 1. Introduction

Urothelial cell carcinoma (UCC) refers to the malignant neoplasm that develops from the transitional epithelia, which includes the tissue in the renal pelvis, ureter, and bladder [1]. Concerning epidemiology, UCC is among the top ten most prevalent cancers in the world [2] and accounts for two percent of deaths resulting from cancer [3]. The risk factors of UCC—age, tobacco consumption, and male gender—could lead to UCC development [4], while the first two of these are related to higher tumor stages [5]. In addition, the different presentation of certain protein expression and genetic variants can also influence the existence of UCC according to previous studies [6,7,8,9,10].

Single-nucleotide polymorphisms (SNPs) have been established in preceding research to have an important role in cancer progression and prognosis [11,12,13]. Located in various regions of DNA, SNPs can alter the production of transcript factors and following translation and gene expression. Recently, the use of SNP assay platforms has enabled the recognition of individual SNPs more conveniently [14]. Certain cancers, such as hepatocellular carcinoma, lung adenocarcinoma, and oral cancer, have been demonstrated with significant SNP alterations [15,16,17,18,19,20]. The SNP and following receptor for advanced glycosylation endproducts also demonstrated a prominent relationship to UCC occurrence [10]. 

Fibroblast growth factor receptor (FGFR) is a protein family that modifies cell proliferation and differentiation, and includes four family members—FGFR1, FGFR2, FGFR3, and FGFR4 [21]. According to a previous study, FGFR4 polymorphism revealed a higher probability of hepatocellular carcinoma, liver cirrhosis, and uterine cervical cancer development [15,22,23]. As FGFR 3, another member of FGFR family, can increase angiogenesis and influence the prognosis in bladder cancer [24], other members in the FGFR family may also influence the outcome or tumor severity of UCC. This possibility has not been fully elucidated.

Herein, we aim to evaluate the association between intronic SNP of FGFR4, including rs2011077, rs351855, rs7708357, and rs1966265, and the clinicopathological characters of UCC with the assistance of polymerase chain reaction (PCR). The correlation of each SNP and the tumor stage and histopathologic grading was analyzed.

## 2. Materials and Methods 

### 2.1. Subject Selection

This study was conducted in the Taichung Veteran General Hospital. Patients diagnosed with UCC in the hospital were enrolled in the study group. The tumor stage and TNM status of all participants in the study group were defined by one urologist, and the histopathologic grades were decided by another pathologist. For pathology features, 102 have CIS, 27 patients had sarcomatoid, and 94 had angiolymphatic components. For clinical features, 272 patients had primary bladder UCC, 156 had primary upper tract UC, 69 had synchronous upper tract UC, and 37 had metachronous upper tract UC during follow up. During the same period, 856 individuals without a malignant history were enrolled as a cancer-free control group with a 1:2 ratio. Venous blood drawing was performed in all the participants and then stored in an ethylenediaminetetra-acetic acid-containing tube. After the procedure, the sample was immediately centrifuged and then preserved in a refrigerator at −80 °C. 

### 2.2. Genomic DNA Extraction and Selection of SNPs

The DNA extraction used a method practiced in the literature [12]. Genomic DNA was extracted from the buffy coat fraction of the blood samples by using the QIAamp DNA blood mini kit (Qiagen, Valencia, CA, USA) according to the manufacturer’s instructions. After the extraction of DNA, four separate SNPs of FGFR4 were selected for analysis, including rs2011077, rs351855, rs7708357, and rs1966265, as these four SNPs were well-defined according to previous research [22].

### 2.3. The Genotyping of SNPs via Real-Time PCR

The genotyping procedure was also used a manner already established before [22]. The allelic discrimination of all the four FGFR4 polymorphisms involving rs2011077 (G/A), rs351855 (C/T), rs7708357 (G/A), and rs1966265 (T/C) was evaluated via the application of ABI StepOne Real-Time PCR System (Applied Biosystems, Foster City, California). Then, the results of real-time PCR were further analyzed by SDS version 3.0 software (Applied Biosystems) using the TaqMan assay technique to increase the specificity of PCR.

### 2.4. Statistical Analysis

SAS version 9.4 (SAS Institute Inc, NC, USA) was employed for all analyses. Descriptive analysis was used to present the basic data, tumor stage, and histopathologic grading. We used the Student t-test to compare the difference in age between the study and control groups, while the Chi-square test was applied to compare the differences of gender and tobacco consumption between the two groups. In the next step, logistic regression was used to calculate the odds ratio (OR) of the different percentages of each SNP between the study and control group, then the adjusted odds ratios (AOR) with their 95% confidence intervals (CIs) were yielded to evaluate the different distributions of FGFR4 SNPs between the UCC and non-UCC individuals by the multiple logistic regression model, which adjusted for age, gender, and tobacco consumption. To investigate the effect of the FGFR4 genotype on the development of UCC more precisely, the logistic regression and associated OR was performed again to evaluate the different distribution of both rs2011077 and rs1966265 in UCC subjects with different tumor stages, TNM status, and histopathologic grading. Due to the small patient numbers in some subgroup, AOR was not performed in the stratified analysis of UCC patients to prevent statistical bias. A p-value of 0.05 or less was regarded to indicate a statistically significant difference using two-tailed probability at 95% CIs.

## 3. Results

A total of 428 patients with UCC were enrolled in the study group, while another 856 non-UCC individuals served as the control group. The age was significantly older in the study group compared to the control group (68.61 ± 11.85 versus 57.18 ± 9.99, P < 0.001). On the other hand, the ratios of gender (269 male and 159 female versus 560 male and 296 female, P = 0.364) and tobacco consumption (130 tobacco consumption versus 298 tobacco consumption, P = 0.131) were similar between the two groups. Generally, the patients with UCC presented with early tumor stage concerning whole tumor stage, tumor T status, lymph node status, and existence of metastasis, while high histopathological grading was observed in the study group. The clinicopathological statuses of UCC in the study group are shown in Table 1.

To evaluate the possible association between FGFR4 polymorphism and the probability of UCC development, we analyzed the genotype frequencies of the four SNPs (rs2011077, rs351855, rs7708357, and rs1966265) between UCC patients and non-UCC participants. To decrease the influence of possible confounding factors, we used the AOR and 95% CI produced via the multiple logistic regression model, which considered the age, gender, and tobacco consumption to evaluate the distribution of SNPs. For the genotype distributions of the different SNPs of FGFR4 between the study and control groups, all SNPs of FGFR4 including rs2011077, rs351855, rs7708357, and rs1966265 did not show a significant difference in the study group compared to the control group after adjustment in multivariable analysis (Table 2). We further divided the UCC population by clinical features (primary bladder UCC and primary upper tract UC). However, no association was observed in the primary bladder UCC and primary upper tract UC subgroup (Supplementary materials Tables S1 and S2).

Next, we analyzed the genotype frequencies of all four FGFR4 SNPs and their association to the clinicopathological characteristics of UCC patients involving whole tumor stage, primary tumor size, lymph node involvement, existence of metastasis, and the degree of histopathologic grading. To achieve this analysis, we categorized the patients in the study group into those with pure homozygous wild-type genetic alleles and those with at least one polymorphic allele of FGFR4 SNP. The results demonstrated that the existence of at least one allele of SNP rs2011077 (TC and CC) was related to a significantly higher tumor stage (odds ratio (OR): 1.751, 95% confidence interval (CI): 1.078–2.846, P = 0.023), larger primary tumor size (OR: 1.637, 95% CI: 1.006–2.662, P = 0.046) and higher histopathologic grading (OR: 1.919, 95% CI: 1.049–3.511, P = 0.032) (Table 3). Moreover, similar trends about prominently higher tumor stage (OR: 1.769, 95% CI: 1.082–2.891, P = 0.022), larger primary tumor size (OR: 1.654, 95% CI: 1.011–2.706, P = 0.044), and higher histopathologic grading (OR: 2.006, 95% CI: 1.096–3.674, P = 0.022) were also observed in those with rs1966265 AG/GG SNP compared to those individuals with the wild-type AA homozygotes (Table 4).

## 4. Discussion

In the current study, there were no significantly different frequencies of FGFR4 SNPs rs2011077, rs351855, rs7708357, and rs1966265 between patients with UCC and controls from Taiwan. However, both SNP rs2011077 and SNP rs1966265 led to an advanced tumor stage mainly resulting from the tumor T status and histopathologic grading. 

The SNPs referred to two distinct nucleotide alleles existing in a significant proportion of the whole human population [14]; these are the most plentiful resource of genetic variations and can locate at the exons, introns, and promoters, as well as 50- and 30-untranslated regions [12,25]. The locus and the sequence of SNPs will influence the effect of such polymorphism on transcription and translation [12]. For instance, the genome expression may be enhanced due to the activation of a promoter, according to previous research [26]. In a clinical aspect, the SNPs can result in the various influences on diseases development including cancers [12,27,28,29,30]. In previous studies, SNPs play an important role in the progression and prognosis of UCC via multiple pathways including WNT1-inducible signaling pathway protein 1-, HOX transcript antisense RNA-, and high mobility group box 1 gene-related routes [6,12,31]. Concerning FGFR, which plays an important role in both the neoplasm transformation and skeletal growth [32], the different expression of FGFR family and the following tumor microenvironment are the main mechanisms of cancer development [33], in which the FGFR inhibitor has been assessed as a therapeutic option for cancer [34,35]. For instance, the Erdafitinib, a pan-FGFR inhibitor, was found to be more effective in managing urinary bladder carcinoma than immune checkpoint inhibitors involving nivolumab and pembrolizumab [32]. The association between UCC and a higher prevalence of alteration of the FGFR family, including FGFR1-4, has been demonstrated before [32]. This may result from the proliferation and angiogenesis function of FGFR [24,36]. The enhancement of the FGFR2 pathway would lead to urothelium proliferation and FGFR1, as well as FGFR3 gene amplification can cause urinary bladder carcinoma [32,37]. Accordingly, FGFR4, a risk factor for various cancers including urinary bladder carcinoma [36], may deteriorate over the course of UCC with different SNPs, which was supported by the results of the current study.

Certain genetic risk factors can lead to the development or progression of UCC, while other genetic variants such as Gly388Arg genotype only lead to a greater risk of recurrence in bladder carcinoma [38]. In the current study, however, the SNPs of FGFR4 only account for a numerically higher AOR in the patients with UCC. There are two explanations for the insignificant results. First, the proliferation of UCC can result from the existence of HOX transcript antisense RNA or other FGFR families [12,24], which the current study failed to evaluate. In addition, about half of the patients in the control group were diagnosed with benign prostatic hyperplasia, in which the proliferation effect of FGFR can lead to the progression of benign prostatic hyperplasia [39]. We speculated that the existence of FGFR4 SNPs rs2011077 and rs1966265 might still have some influence on the development of UCC if we considered the other possible contributor of UCC. In a previous study, the joint effect of the FGFR4 rs351855 GG homozygous genotype and the TP53 mutation resulted in a higher rate of death in those with bladder carcinoma [38]. Consequently, the presence of both the TP53 mutation and the FGFR4 SNP rs351855 included in the current study may also have a significant effect on the UCC development, which needs further validation.

Few studies have evaluated the relationship between the FGFR4 polymorphism and the tumor severity of UCC. In the current study, both the SNP rs2011077 and SNP rs1966265 elevated the risk of UCC progression concerning the tumor stage, tumor size, and the histopathologic grading, while the T status is significantly correlated to the oncological outcome of UCC [40,41,42]. To our knowledge, this is a preliminary experience to find an unrevealed genetic risk factor of progression and prognosis in those with UCC. Moreover, both the SNPs revealed the same trend on the UCC, indicating the homogeneous effects of FGFR4 SNPs on the progression and prognosis of UCC. In previous studies, the SNP rs2011077 has been found to be related to the occurrence of prostate cancer, and SNP rs1966265 influences the therapeutic effect in breast cancer [43,44]. Combined with the current study, these SNPs of FGFR4 may influence a broad spectrum of malignant neoplasm, which needs further investigation.

The non-genetic prognostic factors of UCC include older age and tobacco consumption according to previous articles [5,32]. In the current study, the age was older in the study group while the percentage of tobacco consumption was similar between the two groups. Although age is a significant prognostic factor on the development and progression of UCC and the results in the subgroup analysis might lack stringency without considering the age effect, the age is significantly associated with a higher tumor stage and grade of UCC particularly in those older than 80 years [45]. Since the mean age in the both groups was below 70, the impact of age on this analysis may be minor.

There are still some limitations in the current study. First, the genetic effects of other FGFR family members for UCC such as FGFR3 or WNT1-inducible signaling pathway protein 1 were not considered in the current study. Second, the small numbers of certain subgroups in the intra-group analysis of the study group may lead to some statistical bias. Moreover, we lack the data of five-year survival rate in our study groups, so a more detailed analysis and comparison of FGFR4 polymorphisms to clinical relevance could not be performed. Nevertheless, all the UCC shared the same cell line of transient epithelium; thus, the influence may be minimal in this situation.

## 5. Conclusions

In conclusion, the presence of both FGFR4 SNP rs2011077 and rs1966265 contributed to a higher probability of tumor progression in UCC. A further large-scale prospective clinical trial to evaluate whether the progression and prognosis of UCC is elevated in those with multiple FGFR SNPs compared to a single FGFR SNP is the next research step.

## Figures and Tables

**Table 1:** The distributions of demographical characteristics in 856 controls and 428 patients with urothelial cell carcinoma (UCC).

Variable	Controls (N = 856) n (%)	Patients (N = 428) n (%)	*P*-Value
Age (years)			
Mean ± SD	57.18 ± 9.99	68.61 ± 11.85	<0.001
Gender			
Male	560 (65.4%)	269 (62.9%)	0.364
Female	296 (34.6%)	159 (37.1%)	
Tobacco consumption			
No	560 (65.4%)	298 (69.6%)	0.131
Yes	298 (34.6%)	130 (30.4%)	
Stage			
pTa–pT2		281 (65.7%)	
pT3–pT4		147 (34.3%)	
Tumor T status			
Ta–T2		286 (66.8%)	
T3–T4		142 (33.2%)	
Lymph node status			
N0		378 (88.3%)	
N1 + N2		50 (11.7%)	
Metastasis			
M0		414 (96.7%)	
M1		14 (3.3%)	
Histopathologic grading			
Low grade		53 (12.4%)	
High grade		375 (87.6%)	

**Table 2:** Genotype distributions of fibroblast growth factor receptor 4 (FGFR4) gene polymorphisms in 856 controls and 428 patients with UCC.

Variable	Controls (N = 856) n (%)	Patients (N = 428) n (%)	OR (95% CIs)	AOR (95% CIs)
**rs2011077**				
TT	221 (25.8%)	110 (25.7%)	1.000 (reference)	1.000 (reference)
TC	418 (48.8%)	224 (52.3%)	1.077 (0.813–1.425)	1.267 (0.863–1.860)
CC	217 (25.4%)	94 (22.0%)	0.870 (0.624–1.214)	0.781 (0.487–1.252)
TC + CC	635 (74.2%)	318 (74.3%)	1.006 (0.772–1.312)	1.095 (0.759–1.579)
**rs351855**				
GG	242 (28.3%)	114 (26.6%)	1.000 (reference)	1.000 (reference)
GA	426 (49.7%)	222 (51.9%)	1.106 (0.840–1.457)	1.406 (0.963–2.055)
AA	188 (22.0%)	92 (21.5%)	1.039 (0.744–1.451)	0.982 (0.609–1.582)
GA + AA	614 (71.7%)	314 (73.4%)	1.086 (0.836–1.409)	1.274 (0.886–1.831)
**rs7708357**				
GG	838 (97.9%)	416 (97.2%)	1.000 (reference)	1.000 (reference)
GA	17 (2.0%)	10 (2.3%)	1.185 (0.538–2.611)	1.696 (0.641–4.485)
AA	1 (0.1%)	2 (0.5%)	4.029 (0.364–44.559)	
AG + AA	18 (2.1%)	12 (2.8%)	1.343 (0.641–2.814)	2.039 (0.801–5.190)
**rs1966265**				
AA	221 (25.8%)	107 (25.0%)	1.000 (reference)	1.000 (reference)
AG	420 (49.1%)	226 (52.8%)	0.913 (0.653–1.275)	0.808 (0.500–1.306)
GG	215 (25.1%)	95 (22.2%)	1.111 (0.838–1.473)	1.394 (0.945–2.056)
AG + GG	635 (74.2%)	321 (75.0%)	1.044 (0.799–1.364)	1.187 (0.818–1.723)

CIs: Confidence intervals; OR: Odds ratio with their 95% confidence intervals were estimated by logistic regression models. AOR: Adjusted odds ratio with their 95% confidence intervals were estimated by multiple logistic regression models after controlling for age, gender, and tobacco consumption.

**Table 3:** Distribution frequency of the clinical status and FGFR4 rs2011077 genotype frequencies in 428 UCC patients.

Variable	FGFR4 (rs2011077)			
	TT (%) (n = 110)	TC + CC (%) (n = 318)	OR (95% CIs)	*P* Value
**Stage**				
pTa–pT2	82 (74.5%)	199 (62.6%)	1.000 (reference)	
pT3–pT4	28 (25.5%)	119 (37.4%)	1.751 (1.078–2.846)	0.023 *
**Tumor T status**				
Ta–T2	82 (74.5%)	204 (64.2%)	1.000 (reference)	
T3–T4	28 (25.5%)	114 (35.8%)	1.637 (1.006–2.662)	0.046 *
**Lymph node status**				
N0	101 (91.8%)	277 (87.1%)	1.000 (reference)	
N1 + N2	9 (8.2%)	41 (12.9%)	1.661 (0.779–3.540)	0.185
**Metastasis**				
M0	107 (97.3%)	307 (96.5%)	1.000 (reference)	
M1	3 (2.7%)	11 (3.5%)	1.278 (0.350–4.668)	0.710
**Histopathologic grading**				
Low grade	20 (18.2%)	33 (10.4%)	1.000 (reference)	
High grade	90 (81.8%)	285 (89.6%)	1.919 (1.049–3.511)	0.032 *

* Bold font indicates statistical significance (*p* < 0.05). CIs: Confidence intervals; OR: Odds ratio with their 95% confidence intervals were estimated by logistic regression models.

**Table 4:** Distribution frequency of the clinical status and FGFR4 rs1966265 genotype frequencies in 428 UCC patients.

Variable	FGFR4 (rs1966265)			
	AA (%) (n = 107)	AG + GG (%) (n = 321)	OR (95% CIs)	*P*-Value
**Stage**				
pTa–pT2	80 (74.8%)	201 (62.6%)	1.000 (reference)	
pT3–pT4	27 (25.2%)	120 (37.4%)	1.769 (1.082–2.891)	0.022 *
**Tumor T status**				
Ta–T2	80 (74.8%)	206 (64.2%)	1.000 (reference)	
T3–T4	27 (25.2%)	115 (35.8%)	1.654 (1.011–2.706)	0.044 *
**Lymph node status**				
N0	98 (91.6%)	280 (87.2%)	1.000 (reference)	
N1 + N2	9 (8.4%)	41 (12.8%)	1.594 (0.748–3.400)	0.224
**Metastasis**				
M0	104 (97.2%)	310 (96.6%)	1.000 (reference)	
M1	3 (2.8%)	11 (3.4%)	1.230 (0.337–4.495)	0.754
**Histopathologic grading**				
Low grade	20 (18.7%)	33 (10.3%)	1.000 (reference)	
High grade	87 (81.3%)	288 (89.7%)	2.006 (1.096–3.674)	0.022 *

* Bold font indicates statistical significance (*p* < 0.05). CIs: Confidence intervals. OR: Odds ratio with their 95% confidence intervals were estimated by logistic regression models.

## Supplementary Materials

The following are available online at https://www.mdpi.com/1660-4601/17/1/129/s1. Table S1: Genotype distributions of Fibroblast growth factor receptor 4 (FGFR4) gene polymorphisms in 856 Controls and 272 patients had primary bladder UCC.; Table S2: Genotype distributions of Fibroblast growth factor receptor 4 (FGFR4) gene polymorphisms in 856 Controls and 156 primary upper tract UC.
