# Supplementary material for: Involvement of FGFR4 Gene Variants on the Clinicopathological Severity in Urothelial Cell Carcinoma
Source: Int J Environ Res Public Health. 2019 Dec 23;17(1):129. doi: 10.3390/ijerph17010129 (PMC6982237; doi:10.3390/ijerph17010129)
Supplement: Supplementary file 1 [file ijerph-17-00129-s001.pdf]

Supplementary Materials

Involvement of FGFR4 Gene Variants on the Clinicopathological Severity in Urothelial Cell Carcinoma

Ming-Dow Tsay[1,2,#], Ming-Ju Hsieh[1,3,4,#], Chia-Yi Lee[5,6], Shian-Shiang Wang[1,7,8,9], Chuan-Shu Chen[1,8], Sheng-Chun Hung[1,7,8], Chia-Yen Lin[1,8], Shun-Fa Yang[1,10,*]

Table 1. Genotype distributions of Fibroblast growth factor receptor 4 (FGFR4) gene polymorphisms in 856 Controls and 272 patients had primary bladder UCC.

Variable	Controls (N = 856) n (%)	Patients (N = 272) n (%)	OR (95% CIs)	AOR (95% CIs)
**rs2011077**				
TT	221 (25.8%)	71 (26.1%)	1.000 (reference)	1.000 (reference)
TC	418 (48.8%)	139 (51.1%)	1.035 (0.745–1.438)	0.973 (0.621–1.525)
CC	217 (25.4%)	62 (22.8%)	0.889 (0.603–1.312)	0.815 (0.478–1.391)
TC+CC	635 (74.2%)	201 (73.9%)	0.985 (0.722–1.345)	0.919 (0.602–1.403)
**rs351855**				
GG	242 (28.3%)	74 (27.2%)	1.000 (reference)	1.000 (reference)
GA	426 (49.7%)	139 (51.1%)	1.067 (0.772–1.474)	1.167 (0.751–1.815)
AA	188 (22.0%)	59 (21.7%)	1.026 (0.694–1.518)	0.983 (0.569–1.697)
GA+AA	614 (71.7%)	198 (72.8%)	1.055 (0.777–1.432)	1.111 (0.730–1.691)
**rs7708357**				
GG	838 (97.9%)	266 (97.8%)	1.000 (reference)	1.000 (reference)
GA	17 (2.0%)	5 (1.8%)	0.927 (0.339–2.535)	1.518 (0.463–4.975)
AA	1 (0.1%)	1 (0.4%)	3.150 (0.196–50.540)	---
AG+AA	18 (2.1%)	6 (2.2%)	1.050 (0.413–2.673)	2.013 (0.672–6.029)
**rs1966265**				
AA	221 (25.8%)	68 (25.0%)	1.000 (reference)	1.000 (reference)
AG	420 (49.1%)	142 (52.2%)	0.937 (0.633–1.387)	0.861 (0.498–1.489)
GG	215 (25.1%)	62 (22.8%)	1.099 (0.789–1.531)	1.124 (0.713–1.772)
AG+GG	635 (74.2%)	204 (75.0%)	1.044 (0.763–1.430)	1.034 (0.671–1.594)

CIs: confidence intervals; OR: odds ratio with their 95% confidence intervals were estimated by logistic regression models. AOR: adjusted odds ratio with their 95% confidence intervals were estimated by multiple logistic regression models after controlling for age, gender, and tobacco consumption.

Table S2. Genotype distributions of Fibroblast growth factor receptor 4 (FGFR4) gene polymorphisms in 856 Controls and 156 primary upper tract UC.

Variable	Controls (N = 856) n (%)	Patients (N = 156) n (%)	OR (95% CIs)	AOR (95% CIs)
**rs2011077**				
TT	221 (25.8%)	39 (25.0%)	1.000 (reference)	1.000 (reference)
TC	418 (48.8%)	85 (54.5%)	1.152 (0.763–1.741)	1.113 (0.734–1.689)
CC	217 (25.4%)	32 (20.5%)	0.836 (0.505–1.383)	0.821 (0.494–1.365)
TC+CC	635 (74.2%)	117 (75.0%)	1.044 (0.705–1.547)	1.728 (0.901–3.314)
**rs351855**				
GG	242 (28.3%)	40 (25.6%)	1.000 (reference)	1.000 (reference)
GA	426 (49.7%)	83 (53.2%)	1.179 (0.783–1.774)	1.259 (0.832–1.906)
AA	188 (22.0%)	33 (21.2%)	1.062 (0.645–1.749)	1.096 (0.662–1.813)
GA+AA	614 (71.7%)	116 (74.4%)	1.143 (0.775–1.686)	1.660 (0.888–3.106)
**rs7708357**				
GG	838 (97.9%)	150 (96.2%)	1.000 (reference)	1.000 (reference)

GA	17 (2.0%)	5 (3.2%)	1.643 (0.597–4.521)	1.678 (0.602–4.678)
AA	1 (0.1%)	1 (0.6%)	5.587 (0.348–89.804)	
AG+AA	18 (2.1%)	6 (3.8%)	1.862 (0.727–4.768)	2.055 (0.514–8.226)
**rs1966265**				
AA	221 (25.8%)	39 (25.0%)	1.000 (reference)	1.000 (reference)
AG	420 (49.1%)	84 (53.8%)	0.870 (0.527–1.434)	0.851 (0.513–1.409)
GG	215 (25.1%)	33 (21.2%)	1.133 (0.750–1.713)	1.098 (0.723–1.666)
AG+GG	635 (74.2%)	117 (75.0%)	1.044 (0.705–1.547)	1.728 (0.901–3.314)

CIs: confidence intervals; OR: odds ratio with their 95% confidence intervals were estimated by logistic regression models. AOR: adjusted odds ratio with their 95% confidence intervals were estimated by multiple logistic regression models after controlling for age, gender, and tobacco consumption.
